# Lymphatic malformations in children: retrospective review of surgical and interventional management

**DOI:** 10.1007/s00383-022-05320-x

**Published:** 2022-12-05

**Authors:** Marion Poget, Marco Fresa, Oumama El Ezzi, Guillaume Saliou, Marie-Thérèse Doan, Anthony de Buys Roessingh

**Affiliations:** 1 Department of Pediatric, Children and Adolescent Surgery Service, Lausanne University Hospital (CHUV), Rue du Bugnon 21, 1011 Lausanne, Switzerland; 2 Department of Heart and Vascular Disease, Angiology Service, Lausanne University Hospital (CHUV), Rue du Bugnon 21, 1011 Lausanne, Switzerland; 3 Department of Radiology, Lausanne University Hospital (CHUV), Rue du Bugnon 21, 1011 Lausanne, Switzerland; 4 Present Address: Visceral Surgery Department, Neuchâtel Hospital, Rue de la Maladière 45, 2000 Neuchâtel, Switzerland

**Keywords:** Lymphatic malformation, Surgery, Sclerotherapy, Pediatrics

## Abstract

**Purpose:**

Lymphatic malformations (LMs) are classified as macrocystic, microcystic or mixed. Treatment depends on their characteristics: surgery, sclerotherapy, both combined, systemic treatment or observation. This study aims to analyze the surgical and interventional management of LMs in children over the last two decades in our university hospital.

**Methods:**

Management of children born with LMs between 2000 and 2019 was reviewed. Parameters collected were: malformation characteristics, type of treatment, symptoms, imaging, timing of diagnosis and first treatment, number of interventions, recovery rate, complications and length of stay.

**Results:**

Files of 48 children were reviewed: 27 with macrocystic and 21 with microcystic LMs. There was no statistically significant difference in type of treatment except for combined treatment, more performed in microcystic LMs (*p* = 0.04). Symptoms, imaging, timing of diagnosis and first treatment, number of interventions and complications were not statistically significant. Overall, the number of surgeries was lower than sclerotherapies (*p* = 0.04). Recovery rate after surgery was higher in macrocystic LMs (*p* = 0.01). Complications and length of stay were not statistically significant.

**Conclusion:**

A good rate of recovery was observed when surgery was performed, with no significant increase in complications and length of stay. A prospective study will be determinant to create a decisional algorithm for children with LMs.

## Introduction

Lymphatic malformations (LMs) are benign vascular anomalies composed of dilated lymphatic channels and cysts that affect between 1/200 and 1/4000 live births, equally distributed among males and females [1]. Up to 75% of the LMs are localized in the cervicofacial region, followed by the axilla, chest, gluteus, perineum, retroperitoneum and mediastinum [2]. The exact etiology is unknown, but several theories are hypothesized: connection failure between abnormal endothelial buds and the venous system which it originates from, loss of connection between the buds and the central lymph channels, or pinching out of a proportion of lymphatic channels from the main lymphatic system [3, 4].

LMs are classified as macrocystic (cysts measuring more than 2 cm), microcystic (cysts measuring less than 2 cm) or mixed [5]. The indication and the type of treatment depend on the age of the patient, the localization of the lymphatic malformation, its size, its components (macrocystic or microcystic) and the functional symptoms such as swelling, bleeding, recurrent infection, dysphagia, respiratory distress, or cosmetic deformity [1].

Both ultrasound and magnetic resonance imaging (MRI) determine the extent of the lesions and the anatomic relationship to the adjacent structures [2].

Several treatment modalities are available such as sclerotherapy, surgery, laser coagulation, radiofrequency and systemic treatment for complex, generalized cases [2, 6], yet no treatment algorithms have been established and the patients are treated on an individual basis and need a multidisciplinary approach [3, 4, 7].

This study aims to compare the surgical and radiological (i.e., interventional) management of macrocystic and microcystic LMs in children in terms of number of interventions, recovery rate and postinterventional complications. Evolution of treatment over a 20-year period is also reviewed.

## Material and methods

Demographic and disease-related data of children treated for a lymphatic malformation at Lausanne University Hospital were retrospectively collected from August 2002 to May 2021. Files of children were analyzed according to the type of malformation and its localization. Lesions were classified into macrocystic and microcystic LMs according to the type of cysts predominant (> 50%) inside the lesion, after MRI and/or sonography. Lesions were classified into two groups rather than three (macrocystic, microcystic and mixed LMs) as a result of the limited number of cases included in the study and in order to make a more comprehensible and clearer analysis. Size of the lesions was not taken into account since treatment modality remained unaffected by this characteristic. However, expansion into deeper cavities was considered. Children were categorized into four groups: those treated by surgery, those treated by percutaneous sclerotherapy, those treated by a combination of both and those observed without intervention. All consecutive cases were included in the study.

Initial symptoms, imaging, timing of diagnosis (pre or postnatal), age at first treatment, evolution of treatment over a 20-year period and rate of genetic mutation of PIK3CA were reviewed. Primary endpoints were the number of interventions needed and the response to treatment upon physical examination. Secondary endpoints were based on the rate of postinterventional complications and the length of hospital stay after surgery or sclerotherapy.

The inclusion criteria were pediatric patients with superficial (subcutaneous, intramuscular) or deep simple LMs, according to the International Society for the Study of Vascular Anomalies (ISSVA) classification [8], localized in the cervicofacial region, chest, axilla or limbs. The non-inclusion criteria were an alternative diagnosis such as lipoma, lymphedema, intra-articular cyst, angiolymphoid hyperplasia with eosinophilia, gigantism and lymphangiomatosis (generalized lymphatic anomaly).

Patients regularly meet their surgeon after surgery or interventional treatment at the outpatient clinic of Pediatric Surgery at Lausanne University Hospital. Follow-up lasts for years, as well as for patients being under observation.

Categorical data were quantified as counts and percentages and compared with the use of the chi-square test. Continuous data were displayed as means and standard deviations (SD) or median with interquartile range according to their normal distribution or not and compared using Student’s *t*-test or nonparametric tests, as appropriate. Statistical analyses were carried out by using Prism version 8.4.3 (GraphPad Software, San Diego, California, USA). All statistical tests were two-sided and a *p-*value < 0.05 was considered statistically significant. This study was reviewed and approved by the local Commission on Ethics in Human Research (CER-VD, No. 2021-2685).

## Results

Fifty-eight patients were eligible for the study. Eight of them were excluded due to an alternative diagnosis: 2 had gigantism, 2 had lymphedema, 1 had a lymphangiomatosis, 1 had an intra-articular cyst, 1 had an angiolymphoid hyperplasia with eosinophilia and 1 had a lipoma. Two patients were excluded due to incomplete data: 1 patient with macrocystic LM had missing date and type of sclerotherapy and the type of malformation was not reported in the other patient excluded. There was one loss to follow-up for a patient who moved to France after a period of observation. (No surgical or interventional treatment was performed.) The patient’s data were considered for analysis, except for follow-up.

**Fig. 1:**
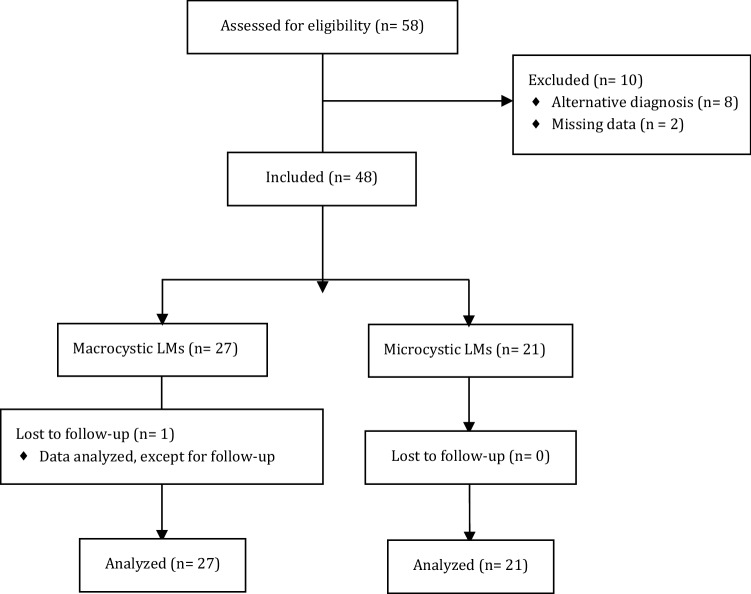
Patient flow diagram

Files of forty-eight children were analyzed: 27 children in the macrocystic LMs group and 21 in the microcystic LMs group. Patient flow diagram is displayed in Fig. 1.

Twenty-four malformations were localized in the cervicofacial area (50%), 11 in the limbs (22.9%), 9 in the thoracic area (18.8%) and 3 in the axilla (6.3%). Localizations of the macrocystic LMs were: 14 in the cervicofacial area (51.9%), 5 in the limbs (18.5%), 6 in the thoracic area (22.2%) and 2 in the axilla (7.4%). Respectively, 11 lesions (52.4%), 6 (28.6%), 3 (14.3%) and 1 (4.8%) in the microcystic LMs group. All but one of the children presented with a superficial lesion, the remaining one having a macrocystic mediastinal LM.

The majority of patients were treated with surgery alone in both groups: 16 in the macrocystic LMs group (59.3%) and 10 in the microcystic LMs group (47.6%) (*p* = 0.42). Two patients in the macrocystic LMs group (including the only patient with a mediastinal lesion) and 1 patient in the microcystic LMs group underwent sclerotherapy alone, representing 7.4% and 4.8%, respectively (*p* = 0.71). A combination of both treatments was administered to 1 patient (3.7%) and 5 patients (23.8%), respectively (*p* = 0.04). Observation was carried out in 8 patients (29.6%) with macrocystic LMs and 5 patients (23.8%) with microcystic LMs (*p* = 0.65). Treatment according to localization is displayed in Table 1.

**Table 1:** Treatment of (a) macrocystic and (b) microcystic malformations according to localization

	Cervicofacial	Thoracic	Axilla	Limb
(a)				
Surgery	8	4	2	2
Sclerotherapy	1	0	0	1
Combination	0	0	0	1
Observation	5	2	0	1
(b)				
Surgery	5	2	1	2
Sclerotherapy	0	1	0	0
Combination	5	0	0	0
Observation	1	0	0	4

Each patient included in the study initially presented with swelling. Two patients (7.4%) with macrocystic LMs and 1 patient (4.8%) with microcystic LM had pain (*p* = 0.71). One patient in each group presented with fever, respectively, 3.7% and 4.8% (*p* = 0.86). In the macrocystic LMs group, 1 patient (3.7%) experienced bleeding (in addition to pain), 1 presented with dyspnea and 1 with dysphagia (in addition to fever). All but one of the patients with pain, bleeding, fever, dyspnea and dysphagia were surgically treated, one of whom underwent a combined treatment. The remaining patient presented pain and was observed.

Sonography was used in 45 patients, respectively, 26 patients (96.3%) and 19 patients (90.5%) in macrocystic LMs and microcystic LMs groups (*p* = 0.41), among whom 36 patients also underwent an MRI (22 patients in macrocystic LMs, i.e., 81.5% and 14 patients in microcystic LMs, i.e., 66.7%, *p* = 0.24). Of these patients, one also underwent a computed tomography (CT). The remaining 3 patients of the study had an MRI alone (3.7% in macrocystic LMs and 9.5% in microcystic LMs).

One (3.7%) macrocystic LM was diagnosed antenatally, against 5 (23.8%) microcystic LMs (*p* = 0.04).

Macrocystic LMs and microcystic LMs were first treated at a median age of 29 months [interquartile range (IQR) 16–59] and 27 months (16–69), respectively (*p* = 0.82). Median age at first treatment was 31 months (IQR 12–65) when surgery alone or as first treatment was used against 20 months (14–22) for sclerotherapy alone or as first treatment (*p* = 0.75).

Macrocystic LMs and microcystic LMs required a mean of 1.3 (SD 0.5) and 1.6 (0.9) surgery alone or combined, respectively, (*p* = 0.26) to remove the malformation. Mean number of sclerotherapies alone or combined was, respectively, 2.3 (0.6) and 2.8 (1.9) in macrocystic LMs and microcystic LMs (*p* = 0.58). The mean number of surgeries required was statistically lower than sclerotherapies (1.4 vs. 2.6), regardless of type of malformation (*p* = 0.04).

Overall response to treatment was assessed on physical examination and divided into good response (i.e., disappearance of the lesion, or stability when observation was carried out) or partial response (i.e., incomplete regression, or growth when observation was carried out).

Twenty patients (74.1%) with macrocystic LMs and 10 patients (47.6%) with microcystic LMs presented a resolution of the malformation upon physical examination (*p* = 0.06); after surgery alone, disappearance of the lesion in 15 patients (93.8%) with macrocystic LMs and in 5 patients (50%) with microcystic LMs (*p* = 0.01); after sclerotherapy alone, a disappearance among the 2 patients with macrocystic LMs and the only one with microcystic LM; after combined treatment, a partial disappearance for the only patient with macrocystic LM and a resolution of the malformation for 3 patients (60%) with microcystic LMs (*p* = 0.27). Observation led to a good evolution (i.e., stability in size) in 3 patients (37.5%) and 1 patient (20%), respectively (*p* = 0.51).

Three patients (15.8%) with macrocystic LMs and 6 patients (37.5%) with microcystic LMs encountered complications regardless of the type of treatment (*p* = 0.08). Table 2 displays primary outcomes (i.e., number of interventions and response to treatment upon physical examination), as well as demographics, type of treatment, clinical presentation, diagnostic imaging, timing of diagnosis and postinterventional complications in macrocystic LMs and microcystic LMs.

**Table 2:** Demographics, type of treatment, clinical presentation, diagnostic imaging, timing of diagnosis, number of interventions, response to treatment upon physical examination and postinterventional complications: comparison between macrocystic and microcystic malformations

	Macrocystic LMs (*n* = 27)	Microcystic LMs ( *n* = 21)	*p*-value
Gender, *n* (%)			
Male	18 (66.7%)	13 (68.9%)	
Female	9 (33%)	8 (31.1%)	
Treatment, *n* (%)			
Surgery alone	16 (59.3%)	10 (47.6%)	0.42
Sclerotherapy alone	2 (7.4%)	1 (4.8%)	0.71
Surgery and sclerotherapy	1 (3.7%)	5 (23.8%)	0.04*
Observation	8 (29.6%)	5 (23.8%)	0.65
Symptoms, *n* (%)			
Swelling	27 (100%)	21 (100%)	
Pain	2 (7.4%)	1 (4.8%)	0.71
Fever	1 (3.7%)	1 (4.8%)	0.86
Bleeding	1 (3.7%)	0 (0%)	
Dyspnea	1 (3.7%)	0 (0%)	
Dysphagia	1 (3.7%)	0 (0%)	
Imaging, *n* (%)			
Sonography	26 (96.3%)	19 (90.5%)	0.41
Sonography alone	4 (14.8%)	5 (23.8%)	0.43
Sonography and MRI	22 (78.6%)	14 (66.7%)	0.24
Antenatal diagnosis, *n* (%)	1 (3.7%)	5 (23.8%)	0.04*
Age at first treatment (months), median (IQR)	29 (16–59)	27 (16–69)	0.82
Number of interventions, mean (SD)			
Surgery alone or combined	1.3 (0.5)	1.6 (0.9)	0.26
Sclerotherapy alone or combined	2.3 (0.6)	2.8 (1.9)	0.58
Response to treatment, *n* (%)			
Overall—good	20 (74.1%)	10 (47.6%)	0.06
Surgery alone—good	15 (93.8%)	5 (50%)	0.01*
Sclerotherapy alone—good	2 (100%)	1 (100%)	
Surgery and sclerotherapy—good	0 (0%)	3 (60%)	0.27
Observation—good	3 (37.5%)	1 (20%)	0.51
Complications (Clavien–Dindo), *n* (%)			
Overall	3 (15.8%)	6 (37.5%)	0.08
I	1 (5.3%)	2 (12.5%)	
II	1 (5.3%)	1 (6.3%)	
III	1 (5.3%)	3 (18.8%)	

*IQR* interquartile range, *SD* standard deviation

^*^Indicates *p* < 0.05

Postoperative complications rate after surgery alone or combined reached 25% (8 patients out of 32) compared to 11.1% (1 out of 9) postinterventional complications after sclerotherapy alone or combined (*p* = 0.37). Clavien–Dindo grade [9] was higher after surgery (grade III) compared to sclerotherapy (grade I): 6 patients (18.8%) had a lymphatic leak after surgery, 4 of them (12.5%) requiring either a puncture or an incision and drainage and the remaining 2 (6.3%) had a local skin infection treated with antibiotics. Two patients (6.3%) experienced a transient facial paralysis. Of all patients treated with sclerotherapy alone or combined, 1 patient (11.1%) presented oral ulcerations after sclerotherapy. No permanent lesions were observed.

Average length of hospital stay was 3 days (SD 4.4) versus 1.3 day (1.2) after surgery and sclerotherapy, respectively (*p* = 0.14). These latter results are displayed in Table 3 along with the number of interventions and postinterventional complications among patients who underwent surgery and/or sclerotherapy.

**Table 3:** Number of interventions, postinterventional length of stay and complications: comparison between surgery and sclerotherapy

	Surgery^†^ (*n* = 32)	Sclerotherapy^†^ (*n* = 9)	*p*-value
Number of interventions, mean (SD)	1.4 (0.7)	2.6 (1.5)	0.04*
Complications (Clavien–Dindo), *n* (%)	8 (25%)	1 (11.1%)	0.37
I	2 (6.3%)	1 (11.1%)	
II	2 (6.3%)	0 (0%)	
III	4 (12.5%)	0 (0%)	
Postinterventional stay, mean (SD)	3 (4.4)	1.3 (1.2)	0.14

^†^Alone or combined

^*^Indicates *p* < 0.05

Twenty-eight patients (58.3%) were still followed when the study ended: 13 in the macrocystic LMs group (46.4%) and 15 in the microcystic LMs group (71.4%). One patient was lost to follow-up after moving to France. Among the patients followed, two patients with macrocystic LMs were in treatment when the study ended and none with microcystic LM. The remaining 26 patients were under observation. Most patients whose follow-up ended had been treated with surgery alone (14 in 19 patients, 73.7%), 2 patients (10.5%) had had a combined treatment and 3 patients (15.8%) had been observed. None of them had had sclerotherapy alone. At the completion of the study, the last patient stopped being followed had had surgery 4 years earlier. The other children stopped being followed had had a treatment minimum 9 years earlier.

**Fig. 2:**
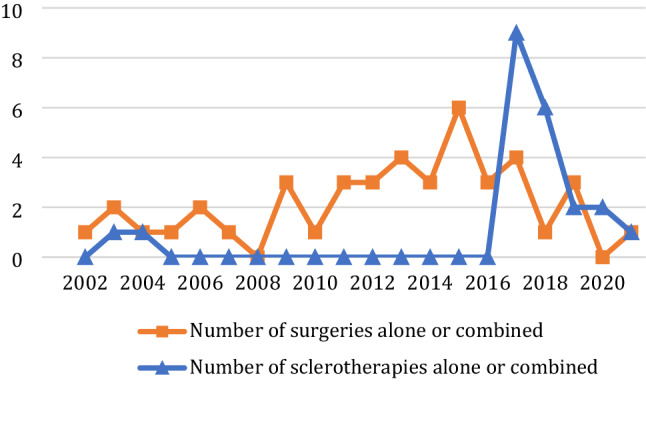
Number of surgical and radiological treatments performed from 2002 to 2021

Use of surgical and radiological treatment evolved over this 20-year period. Figure 2 shows that surgery was constantly used unlike radiological treatment, which was increasingly performed since 2017.

**Fig. 3:**
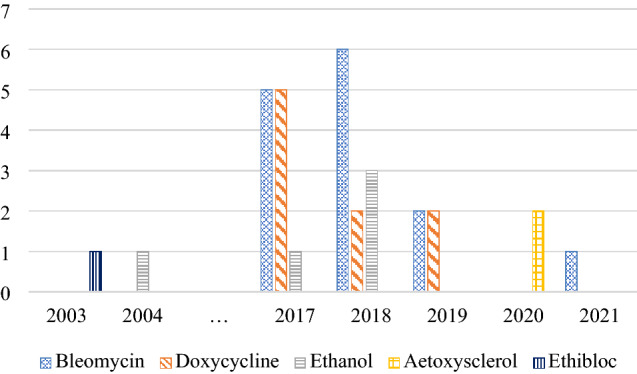
Use of sclerosing agents over the years (2003–2021)

Use of agents for sclerotherapy also evolved over this 20-year period, as shown in Fig. 3. Ethibloc was used one time in 2003, and ethanol was administered five times between 2004 and 2018, doxycycline used nine times from 2017 to 2019 and aetoxysclerol two times in 2020. Bleomycin was the most popular agent given 14 times from 2017 to 2021.

PIK3CA mutation was searched one time and turned out positive in a patient with microcystic LM. This patient had both sclerotherapy and surgery with a partial response.

## Discussion

In general practice, no single modality of treatment is effective and multimodal therapy is often necessary, especially in patients with widespread disease and for whom therapeutic options are often palliative [10]. That is why a multidisciplinary team was created in 2015 at Lausanne University Hospital, which includes a pediatric surgeon, a plastic surgeon, an interventional radiologist, a dermatologist and an angiologist. The presence of the latter is essential for the patients’ follow-up throughout adulthood.

Percutaneous sclerotherapy under ultrasound or fluoroscopy is nowadays considered as the first-line treatment of LMs and has a greater success with macrocystic LMs [2–4, 11–13]. However, studies assessing intralesional bleomycin efficacy for microcystic LMs have shown a satisfying outcome based on imaging [14, 15]. A new sclerotherapy technique called the lymphographic-like technique recently came up to specifically address microcystic LMs components < 3 mm. It consists of inserting 4–8 needles into the lesion and infusing bleomycin at a very slow flow rate, with good outcomes [14]. This slow infusion of sclerosing agent was also found safe and effective by Lee et al. [16].

Many agents have been described, for instance doxycycline, bleomycin, absolute ethanol, betadine, sodium tetradecyl sulfate, polidocanol, OK-432 and alcoholic zein solution [1, 2, 10, 12, 17]. Ethanol stopped being used due to a high complication rate (ulceration, nerve injury and systemic toxicity) [3, 18].

In our study, bleomycin and doxycycline were the most commonly used agents in sclerotherapy, as seen in the literature [1, 2]. Bleomycin is the preferred agent due to its minimal inflammatory reaction and the absence of pain during injection [15].

In 2021, after completion of the study, started in our institution the use of polidocanol in foam or liquid form for the treatment of LMs. This molecule is widely described and known to have a low complication rate and a good efficacy profile, as well as a painless injection [19]. Its good visibility under ultrasound guidance allows to perform the procedure without any radiation exposure, and since the molecule is painless it is suitable for a completely outpatient setting [19, 20]. The foam form relatively reduces the dose of sclerosing agent used, and thus, it has fewer potential risks of complications [20]. Furthermore, microfoam increases the lasting surface contact, resulting in a more efficient endothelial cytolytic effect [20].

Surgery was the most common treatment given for microcystic LMs in our study, as seen in common practice [3, 21]. It was associated with a good recovery rate among patients with macrocystic LMs. Half of patients with microcystic LMs responded partially, as expected, due to the infiltrative nature of these lesions and their relationship to important neurovascular adjacent structures, especially in the cervicofacial region [7, 22–25]. The surgical approach is performed for severe lymphatic disorders unresponsive to other therapies but is still associated to a high rate of recurrence [10]. Laser therapy and radiofrequency ablation can be considered as other therapeutic modalities in microcystic LMs [2].

Our results contrast with data found in the literature regarding macrocystic LMs: The majority of these lesions were treated with surgery alone, whereas sclerotherapy is indicated as a first-line treatment in this type of malformation [3, 10–12]. The divergent practices between our department and the data found in the literature can be partly explained by the creation of the multidisciplinary team in 2015. Until 2014, the pediatric surgeon used to see the children with LMs on his own and to schedule an operation. Since 2015, discussion is multidisciplinary and leads to a change in treatment paradigm with combination of sclerotherapy and surgery.

It is pointed that the number of surgeries required was significantly lower compared to the number of sclerotherapies in our institution to achieve recovery, as seen in the literature [3, 4, 15, 26]. A systematic review carried out in 2012 did not show a superiority of surgery over sclerotherapy in terms of recovery rate for cervicofacial lesions [17]. A meta-analysis conducted in 2019 showed a complete recovery rate for cervicofacial macrocystic LMs and microcystic LMs of 50.5% after a sclerotherapy treatment [12]. Macrocystic LMs tended to respond better than microcystic LMs or mixed LMs, with a recovery rate of, respectively, 53.1%, 35.1% and 31.1% [12]. Observation led to a partial response in most cases in our study, as a spontaneous decrease in size is rarely encountered [11].

Lesions localized in the cervicofacial area can lead to an airway obstruction and dysphagia [4]. Life-threatening complications such as airway compromise may require either an orotracheal intubation or a tracheostomy before treatment, including ex utero intrapartum treatment (EXIT) procedure when prenatal diagnosis is made [2, 4, 27]. Recent studies showed that sclerotherapy was a safe and effective procedure to rapidly reduce the size of the lesion [28, 29]. Surgery can be also considered, as well as systemic treatment with Rapamycin (Sirolimus^®^) in spontaneously breathing neonates [30].

Recently, mTOR inhibitors showed the promising results in the management of vascular anomalies. mTOR is a serine threonine kinase regulated by phosphoinositide 3 kinase (PI3K) and protein kinase B (Akt) [1]. The PI3K/Akt/mTOR pathway is the basis for cell growth and proliferation; it also increases the expression of the vascular endothelial growth factor (VEGF) regulating angiogenesis and lymphangiogenesis. mTOR inhibitors directly inhibit mTOR, blocking downstream protein synthesis and presenting antitumoral and antiangiogenic effect [1, 31–33]. Rapamycin (Sirolimus^®^) is one of the best-known mTOR inhibitors. Resistant complex lymphatic anomalies with visceral and bony adverse effects have shown good response to Sirolimus^®^ [34, 35]. A somatic mosaic activating mutation in PIK3CA leads to a tissular overgrowth associated with vascular anomalies. The presence of such a mutation must be assessed in order to offer an appropriate treatment like alpelisib, also an mTOR inhibitor, currently undergoing a testing phase with the promising results [36].

**Fig. 4:**
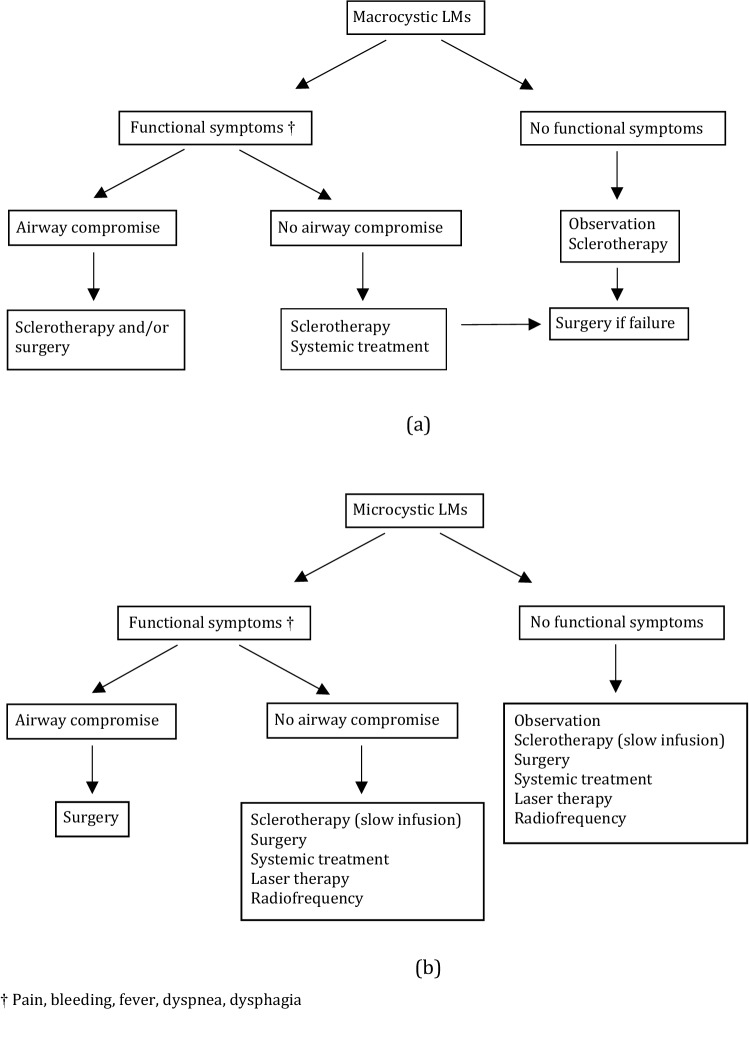
Proposal of treatment decision making algorithm for lymphatic malformations according to malformation type and symptoms: **a** algorithm for macrocystic malformations; **b** algorithm for microcystic malformations. ^†^Pain, bleeding, fever, dyspnea, dysphagia

A proposal of treatment algorithm according to malformation type and symptoms based on our data and the data found in the literature is displayed in Fig. 4.

The complications rates, their severities and the length of postoperative stay after either surgery or sclerotherapy were not statistically significant, which contrasts with the data found in the literature. Indeed, these latter outcomes have been found higher when surgery is performed compared to sclerotherapy [37].

Complications after surgery include injuries of the facial nerve and hypoglossal nerve, seroma, tissue defects, bleeding, infection, Horner syndrome and development of vesicles on the incision site [2, 37, 38]. The rate of surgical complications encountered in our study was close to the data found in the literature. The reported complication rates after surgery range from 12 to 33% for lesions localized in the cervicofacial area, chest and limbs [39], which approximates to our results.

Permanent lesions after sclerotherapy for cervicofacial malformations are reported at a rate of 1.2% (facial palsy, Horner syndrome) [13]. Burrows et al. reported a rate of 2% of major complications for malformations localized in head and neck, chest and limbs [37]. Temporary complications (swelling, inflammation, transient superficial necrosis, bleeding, transient nerve injury) seem to occur in 14% of cases after sclerotherapy for cervicofacial malformations [13], and in 10% of cases when cervicofacial, thoracic or limb lesions are considered [37]. These rates are similar to the results of our study.

Several limitations to this study can be identified. First, the study is designed in a retrospective way and is monocentric. Second, a limited sample of patients is included, not allowing to compare the management of malformations according to their localizations. Furthermore, lesions were classified into two groups rather than three (macrocystic, microcystic and mixed LMs) as a result of the limited number of cases included in the study.

The statistical robustness regarding the recovery rate is also affected. The head of Plastic Pediatric Surgery department influenced the choice of treatment, leading to a non-use of sclerotherapy for 13 years and a consecutive bias.

This study emphasizes the need of a multidisciplinary approach to treat patients with LMs. Indeed, although it is commonly accepted that macrocystic LMs are treated by sclerotherapy, the treatment of microcystic LMs remains debated,

and growing evidence shows the efficiency of sclerotherapy to treat these lesions.

A prospective study will be a determining factor for the establishment of a treatment algorithm for children with LMs.

## Data Availability

The data presented in this study are available on request from the corresponding author.
